# Seasonality of Bacterial Strains in Diabetic Foot Osteomyelitis: Implications for Empiric Antibiotic Therapy in a Temperate Region with Distinct Seasons

**DOI:** 10.3390/jcm15052064

**Published:** 2026-03-09

**Authors:** Chung-Shik Shin, Dong-whee Kim, Jong-kil Kim, Tae-ho Kim

**Affiliations:** Department of Orthopaedic Surgery, Presbyterian Medical Center, Jeonju 54987, Republic of Korea; zsnew@hanmail.net (C.-S.S.); cocaccola@naver.com (D.-w.K.); ullicease@naver.com (J.-k.K.)

**Keywords:** diabetic foot, osteomyelitis, seasonality, *Pseudomonas aeruginosa*, amputation

## Abstract

**Background**: Diabetic foot osteomyelitis (DFO) is a severe complication requiring effective empiric antibiotic therapy to prevent amputation. While global guidelines suggest tailoring therapy based on climate zones, limited data exist regarding seasonal variations within a single region experiencing distinct seasonal extremes. This study investigated whether the bacterial etiology of DFO differs significantly between the hot, humid summer and the cold, dry winter in the Republic of Korea. **Methods**: We conducted a retrospective cohort study of 85 patients with DFO who underwent lower extremity amputation between January 2018 and October 2024. Patients were categorized into Summer (July–August) and Winter (December–January) groups. Deep tissue or bone specimens were analyzed to compare pathogen prevalence. **Results**: A total of 85 patients were included (Summer: *n* = 45; Winter: *n* = 40). While *Staphylococcus* species were the most common pathogens overall (30.6%), a seasonal shift was observed. The proportion of Gram-negative isolates was higher in Summer (50.7%) compared to Winter (35.1%), representing a notable clinical trend (*p* = 0.080). Specifically, *Pseudomonas aeruginosa* and *Escherichia coli* were more frequently isolated during the summer months. Furthermore, polymicrobial infections were more prevalent in Summer (62.2%) compared to Winter (45.0%), although this did not reach statistical significance (*p* = 0.111). **Conclusions**: The microbiological profile of DFO exhibits seasonal variations. The observed trend toward an increased prevalence of Gram-negative and polymicrobial infections during the Korean summer suggests that empiric antibiotic guidelines should be dynamic rather than static.

## 1. Introduction

Diabetic foot infection (DFI) is one of the most serious and costly complications of diabetes mellitus and remains a leading cause of non-traumatic lower extremity amputation worldwide. DFI is associated with a 5-year mortality rate comparable to many common cancers, and factors such as peripheral neuropathy, ischemia, and deep tissue involvement significantly exacerbate disease severity. Furthermore, recent evidence highlights that geographical and climatic variations not only alter the pathogen spectrum but also affect antimicrobial resistance (AMR) rates, with tropical regions often reporting higher rates of multidrug-resistant Gram-negative infections and increased disease severity [1,2]. Among the spectrum of DFIs, diabetic foot osteomyelitis (DFO) represents an advanced and limb-threatening condition associated with prolonged hospitalization, repeated surgical interventions, and high morbidity [1,3]. Early initiation of appropriate antibiotic therapy is therefore critical to improving clinical outcomes.

Empiric antibiotic selection for DFO has traditionally been guided by microbiological data from Western countries, where Gram-positive cocci—particularly *Staphylococcus aureus*—predominate [2]. Consequently, international guidelines generally recommend empiric regimens primarily targeting Gram-positive organisms in temperate climates [2]. However, increasing evidence suggests that the microbiology of DFI varies considerably according to geography, climate, and environmental conditions [4,5].

Studies from tropical and subtropical regions have consistently reported a higher prevalence of Gram-negative bacteria and polymicrobial infections in DFIs compared with reports from Western countries [4,5]. Recent epidemiological studies further indicate that climatic factors such as ambient temperature and humidity may influence bacterial growth, virulence, and transmission dynamics, particularly among Gram-negative organisms [6,7].

Despite these observations, climate has often been treated as a static regional characteristic rather than a dynamic factor that fluctuates within the same geographic area over time. This simplification may obscure clinically relevant temporal variations in pathogen distribution and lead to empiric antibiotic strategies that are insufficiently responsive to changing environmental conditions.

The Republic of Korea provides a unique environment for examining this phenomenon. Although it is classified as a temperate country, it experiences marked seasonal extremes, including hot, humid summers and cold, dry winters. Such marked intra-regional climatic variability offers a natural model to explore whether seasonal environmental changes are associated with shifts in the microbiological characteristics of DFO within a single healthcare system.

Despite these climatic variations, limited data are available regarding whether seasonal changes influence the causative pathogens of DFO and whether empiric antibiotic strategies should be adjusted accordingly.

Therefore, the objective of this study was to investigate seasonal differences in bacterial strains causing DFO in the Republic of Korea. We hypothesized that infections occurring during summer months would demonstrate a higher prevalence of Gram-negative and polymicrobial pathogens compared with winter infections.

## 2. Materials and Methods

### 2.1. Study Design and Setting

This study was designed as a retrospective cohort analysis and was conducted at a secondary academic medical center located in Jeonju, Republic of Korea. This region is characterized by a humid continental climate with hot summers and cold winters. The study protocol adhered to the ethical guidelines of the Declaration of Helsinki and was approved by the Institutional Review Board (No. PMC 2024-11-054-005).

The retrospective cohort design was selected to enable the analysis of real-world clinical data accumulated over multiple years. This approach allowed for the inclusion of consecutive patients treated under routine clinical conditions, thereby enhancing the external validity of the findings. By spanning multiple calendar years, the study design also reduced the likelihood that observed seasonal differences were attributable to short-term institutional or practice-related changes.

### 2.2. Patient Selection and Data Collection

A total of 314 patients diagnosed with DFI underwent surgical treatment between January 2018 and October 2024. To clearly compare the effects of climate extremes, we initially selected 93 patients who were operated on during the defined peak summer and winter months. Among them, 8 patients were excluded because their lesions did not meet the severity inclusion criteria of Wagner grade 3, 4, or 5 [8]. Consequently, a total of 85 patients met the final inclusion criteria (Summer: *n* = 45; Winter: *n* = 40). Non-amputated patients were excluded from this study because superficial swab cultures are highly susceptible to contamination and may not accurately reflect the true pathogens causing deep bone infections.

The use of MRI as a mandatory diagnostic criterion was intended to improve diagnostic accuracy and ensure a consistent case definition across the study population. MRI findings were interpreted in conjunction with clinical signs and intraoperative findings to minimize false-positive diagnoses of osteomyelitis. Restricting inclusion to surgically treated cases ensured the availability of deep tissue or bone specimens, which are more reliable for microbiological analysis than superficial swab cultures.

Climate Definitions: Based on climate data from the Korea Meteorological Administration for the Jeonju region, “Summer” was defined as July and August (highest average temperatures, mean 26.9 °C), and “Winter” was defined as December and January (lowest average temperatures, mean 1.4 °C).

These months were selected to represent periods of maximal climatic contrast, thereby increasing the sensitivity of the analysis to detect season-related differences. Intermediate seasons were intentionally excluded to reduce misclassification and overlap between climatic conditions.

In addition to seasonal categorization, patient management protocols at our institution remained consistent throughout the study period. Indications for surgical intervention, perioperative wound care, and microbiological sampling were standardized and did not differ according to season. This consistency reduces the likelihood that observed microbiological differences were driven by variations in clinical practice rather than environmental factors. Furthermore, antibiotic stewardship policies at the institution did not incorporate season-based adjustments during the study period, allowing for an unbiased assessment of natural seasonal variation in pathogen distribution.

### 2.3. Surgical Procedure and Microbiological Sampling

All surgical procedures were performed by a single experienced orthopedic surgeon. To minimize contamination from skin flora, the following procedures were performed:

1. The foot was cleansed with povidone–iodine.
2. Debridement of necrotic soft tissue was performed first.
3. A separate set of sterile instruments was used to obtain deep tissue or bone biopsy specimens from the infected focus.
4. Specimens were immediately placed in anaerobic transport medium and transported to the laboratory within 1 h.

Standardizing the surgical and sampling technique across all cases was intended to reduce procedural variability that could influence culture results. Deep tissue or bone specimens were preferentially obtained from areas with macroscopic evidence of infection, such as purulence, necrotic bone, or cortical destruction. This strategy increased the likelihood that isolated organisms represented true causative pathogens rather than contaminants.

### 2.4. Microbiological Identification

Specimens were inoculated onto blood agar, MacConkey agar, and chocolate agar plates for bacterial isolation. Additionally, Sabouraud dextrose agar was utilized to screen for fungal pathogens. Cultures were incubated at 35–37 °C for 72 h. Bacterial identification and antimicrobial susceptibility testing (AST) were performed using an automated microbial identification system (the VITEK^®^ 2 system, bioMérieux, Craponne, France) strictly following the Clinical and Laboratory Standards Institute (CLSI) guidelines. The combination of culture media was selected to facilitate the growth of both Gram-positive and Gram-negative organisms commonly implicated in diabetic foot infections.

### 2.5. Data Collection and Statistical Analysis

Patient demographics, including age, sex, and body mass index, were recorded. Comorbidities such as chronic kidney disease and peripheral vascular disease were also collected. Microbiological data were categorized according to Gram stain characteristics and bacterial species.

Continuous variables are expressed as means ± standard deviation and compared using Student’s *t*-test. Categorical variables were compared using the chi-square test. A *p*-value < 0.05 was considered statistically significant. Statistical analyses were performed using SPSS version 18.0 (SPSS Inc., Chicago, IL, USA). The statistical analysis plan was predefined to focus on comparisons between seasonal groups. No post hoc subgroup analyses were performed to avoid overinterpretation of the data. This analytical approach was chosen to maintain clarity and reduce the risk of spurious associations.

## 3. Results

### 3.1. Demographic Characteristics

There were no significant differences in baseline characteristics between the Summer and Winter groups (Table 1). The mean age was 45 ± 13.9 years in the Summer group and 40 ± 12.5 years in the Winter group (*p* = 0.245). Both groups showed a male predominance, but the sex ratio did not differ significantly (*p* = 0.270). Comorbidities such as chronic kidney disease and peripheral vascular disease were also comparable. The similarity of baseline characteristics suggests that seasonal differences in microbiological findings are unlikely to be explained by demographic or clinical confounders.

### 3.2. Microbiological Distribution

*Staphylococcus* species (including *S. aureus* and coagulase-negative staphylococci) were the most frequently identified pathogens in both seasons, accounting for 30.6% (38/124) of all isolates. However, the proportion of *S. aureus* was higher in Winter (28.1%, 16/57) compared to Summer (16.4%, 11/67) (Table 2). Additionally, no fungal pathogens were isolated from the deep tissue specimens in our final cohort.

**Figure 1:**
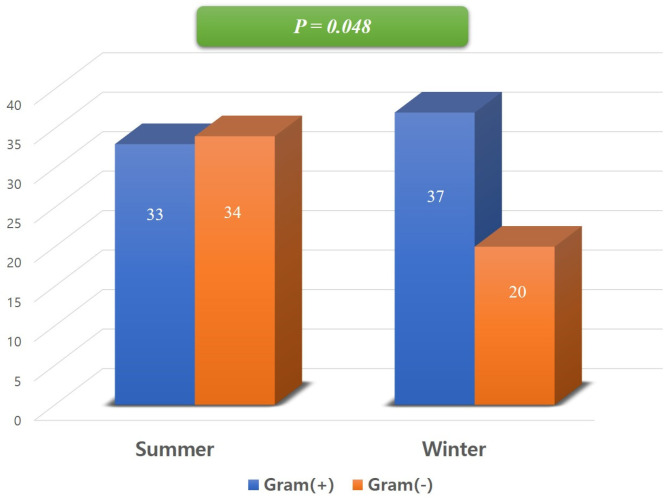
The ratio of Gram-positive to Gram-negative bacteria between summer and winter. The proportion of Gram-negative bacteria was significantly higher in summer compared to winter.

A notable seasonal difference was found in the Gram stain characteristics. The proportion of Gram-negative bacteria showed a strong trend toward being higher in the Summer group (50.7%, 34/67) compared to the Winter group (35.1%, 20/57) (X^2^ = 3.068, *p* = 0.080) (Figure 1). Among Gram-negative bacteria, *Pseudomonas aeruginosa* (11.9%, 8/67) and *Escherichia coli* (11.9%, 8/67) were the most common in Summer.

### 3.3. Polymicrobial Infections

**Figure 2:**
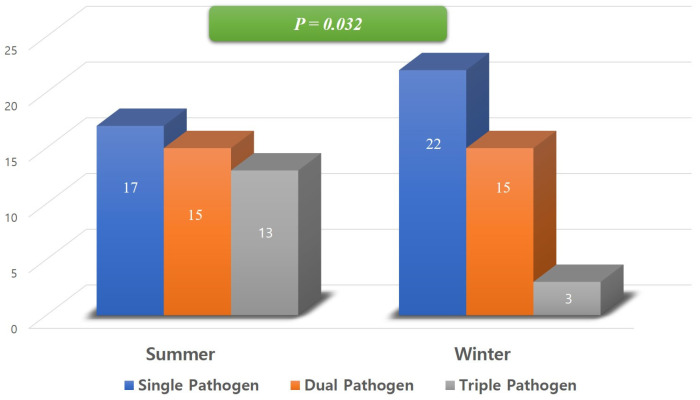
The number of pathogens between summer and winter. Multiple pathogen infections were more common in summer compared to winter.

The complexity of infections also varied by season. Polymicrobial infections (≥2 pathogens) were identified in 54.1% (46/85) of the total cohort. The rate of polymicrobial infection was higher in the Summer group (62.2%, 28/45) compared to the Winter group (45.0%, 18/40), although the difference was not statistically significant (X^2^ = 2.533, *p* = 0.111) (Figure 2).

## 4. Discussion

This study demonstrates a distinct seasonal trend in the etiology of diabetic foot osteomyelitis in the Republic of Korea. We found that during the hot and humid summer, the prevalence of Gram-negative pathogens and polymicrobial infections noticeably increases compared to the cold winter, although these differences did not reach strict statistical significance in our cohort.

This finding highlights that microbial epidemiology in DFO is not static and may fluctuate in response to environmental conditions, even within the same healthcare setting. Such variability challenges the assumption that pathogen distributions in temperate regions remain stable throughout the year.

These findings suggest that seasonal climatic factors may influence pathogen distribution beyond traditional geographic classifications. Previous studies from cool temperate regions, such as Scotland, have reported a consistent predominance of *Staphylococcus aureus* throughout the year [2]. In contrast, studies from tropical and subtropical regions consistently demonstrate a higher burden of Gram-negative pathogens in diabetic foot infections [4,5]. Korea’s climate appears to alternate between these profiles depending on the season, with summer infections resembling those reported in tropical settings.

The present results suggest that Korea occupies an intermediate epidemiological position, in which seasonal extremes temporarily shift the microbial spectrum toward patterns more commonly observed in tropical climates. This seasonal convergence may have important implications for empiric antibiotic strategies that are currently based primarily on geographic location.

Traditionally, the microbiology of diabetic foot infections has been characterized by the predominance of Gram-positive cocci, particularly *Staphylococcus aureus*, which has formed the basis for empiric antibiotic recommendations in many international guidelines [2,3]. However, accumulating evidence indicates that this paradigm does not uniformly apply across different geographic or environmental contexts. Studies from tropical and subtropical regions have consistently reported a higher burden of Gram-negative organisms and polymicrobial infections in diabetic foot infections [4,5]. Our findings suggest that a similar microbiological profile may emerge seasonally in temperate regions during periods of high temperature and humidity. Importantly, this seasonal shift does not negate the continued clinical relevance of Gram-positive pathogens but rather underscores the need for broader initial coverage during specific high-risk periods. Failure to account for such shifts may lead to inadequate empiric therapy, particularly in severe or limb-threatening infections.

The observed seasonal increase in Gram-negative bacteria during summer is biologically plausible. Experimental and epidemiological studies have demonstrated that ambient temperature can influence bacterial growth rates, virulence, and survival, particularly among Gram-negative organisms [7]. Temperature-dependent modifications of lipid A in the outer membrane of Gram-negative bacteria have been shown to enhance bacterial fitness and pathogenicity under warmer conditions [9,10]. In addition, humid environments may facilitate bacterial persistence in wounds and promote colonization by water-associated organisms such as *Pseudomonas aeruginosa*, which was frequently isolated in the Summer group in our cohort.

In diabetic foot wounds, these environmental effects may be amplified by local factors such as impaired perfusion, neuropathy, and reduced immune responsiveness. Together, these factors may create a permissive environment for Gram-negative bacteria to proliferate during warmer months.

Another notable finding of this study is the significantly higher prevalence of polymicrobial infections during the summer months. Polymicrobial DFO is clinically relevant because it is often associated with increased treatment complexity, prolonged antibiotic courses, and poorer outcomes. Environmental conditions favoring bacterial proliferation may increase the likelihood of simultaneous colonization by multiple organisms, thereby promoting polymicrobial biofilm formation. Recent studies have emphasized that polymicrobial infections in diabetic foot disease represent a distinct clinical entity requiring broader empiric coverage and careful antimicrobial stewardship [6].

From a pathophysiological perspective, polymicrobial biofilms exhibit synergistic interactions that enhance bacterial survival and resistance to antimicrobial agents. These interactions may partially explain the greater therapeutic challenges encountered during the summer months.

From a practical standpoint, incorporating seasonality into empiric antibiotic decision-making does not necessarily imply routine use of broad-spectrum agents year-round. Rather, it supports a risk-stratified approach in which broader Gram-negative coverage is selectively considered during periods associated with higher prevalence, such as the summer months identified in this study. This strategy aligns with antimicrobial stewardship principles by balancing the risks of undertreatment against unnecessary antibiotic exposure. Seasonal epidemiological awareness may therefore complement existing guideline frameworks and enhance their applicability to regions with pronounced climatic variability.

Current international guidelines, including the IWGDF/IDSA recommendations, generally advise empiric antibiotic regimens targeting Gram-positive organisms in temperate climates, with additional Gram-negative coverage reserved for patients in tropical or subtropical regions or those with severe infections [2]. However, our findings suggest that such recommendations may require a more nuanced application. In regions like the Republic of Korea, where climatic conditions fluctuate markedly by season, geographic classification alone may be insufficient to guide empiric therapy. Instead, seasonal climatic factors should be considered when selecting initial antibiotic regimens for DFO, particularly during the summer months when Gram-negative and polymicrobial infections are more prevalent.

A season-adapted approach to empiric antibiotic selection may therefore represent a more precise and context-sensitive strategy. Specifically, broader Gram-negative coverage during summer months may be justified, particularly in patients presenting with advanced infection or requiring surgical intervention.

This study has several limitations that warrant consideration. First, its retrospective design limits causal inference. Second, while baseline severity was somewhat controlled, as all patients had advanced infections (Wagner grade ≥ 3) requiring amputation, other potential confounding variables, such as wound chronicity and the exact timing of pre-hospital medical interventions, could not be perfectly adjusted for. Third, because our primary endpoint was the cross-sectional microbiological profile, detailed longitudinal clinical outcomes, differences in disease severity by specific pathogens, and the appropriateness of preoperative antimicrobial therapy were not systematically evaluated. Future multicenter, prospective studies incorporating antimicrobial resistance profiles, treatment regimens, and patient outcomes are needed to confirm these findings and to establish evidence-based, season-specific empiric antibiotic strategies.

Despite these limitations, the strengths of this study include the use of intraoperative deep tissue and bone cultures, which are considered the gold standard for microbiological diagnosis in DFO and minimize the risk of contamination associated with superficial swab cultures [11,12]. By focusing on clearly defined seasonal extremes, this study provides clinically relevant evidence that challenges the assumption of static microbiological patterns in temperate regions.

Overall, these findings contribute to a growing body of literature advocating for more individualized and adaptive approaches to infection management. Incorporating seasonal epidemiological data into clinical decision-making may enhance the precision of empiric antibiotic therapy and ultimately improve patient outcomes.

## 5. Conclusions

This study demonstrates that the microbiological profile of diabetic foot osteomyelitis (DFO) exhibits significant seasonal variation even within a temperate climate. The key innovative aspect of this work is the demonstration that climatic seasonality—specifically hot and humid summer conditions—can shift the bacterial spectrum of DFO toward a higher prevalence of Gram-negative and polymicrobial infections within a single geographic region traditionally classified as temperate. These findings challenge the conventional assumption that empiric antibiotic strategies in temperate regions can remain static throughout the year.

From a clinical perspective, our results support a dynamic, season-adapted approach to empiric antibiotic selection for DFO. While standard Gram-positive-focused regimens may be appropriate during winter months, broader empiric coverage targeting Gram-negative pathogens, including *Pseudomonas aeruginosa*, should be considered during summer, particularly in severe or limb-threatening infections.

Several limitations should be acknowledged. First, the retrospective, single-center design may limit causal inference and generalizability. Furthermore, the strict inclusion criteria resulted in a relatively small sample size, which likely limited the statistical power to detect significant differences in some microbiological variables. Second, this study did not evaluate antimicrobial resistance patterns in relation to seasonal variation, nor did it directly assess clinical outcomes associated with empiric antibiotic adequacy. Third, potential confounding factors such as prior antibiotic exposure and wound care practices could not be fully controlled.

Future research should focus on prospective, multicenter studies to validate these findings across diverse temperate regions. Incorporating antimicrobial resistance profiles, treatment strategies, and clinical outcomes will be essential to determine whether season-adapted empiric antibiotic protocols improve limb salvage and patient prognosis. Ultimately, integrating climatic and seasonal factors into infection management algorithms may contribute to more precise and context-sensitive care for patients with diabetic foot osteomyelitis.

## Figures and Tables

**Table 1:** Demographic and clinical characteristics of patients with diabetic foot osteomyelitis in Summer vs. Winter.

Characteristic	Summer (*n* = 45)	Winter (*n* = 40)	*p*-Value
Age (years, Mean ± SD)	45 ± 13.87	40 ± 12.47	0.245
Gender (Male/Female)	35/10	35/5	0.270
BMI (kg/m^2^, Mean ± SD)	24.50 ± 9.87	23.67 ± 10.56	0.259
Chronic Kidney Disease (*n*, %)	21 (46.7%)	19 (47.5%)	0.587
Peripheral Vascular Disease (*n*, %)	28 (62.2%)	25 (62.5%)	0.359

**Table 2:** Comparison of bacterial isolates from diabetic foot osteomyelitis lesions in Summer and Winter.

Pathogen	Summer (*n* Isolates)	Winter (*n* Isolates)
Gram-positives	33 (49.3%)	37 (64.9%)
*Staphylococcus aureus* (total)	11 (16.4%)	16 (28.1%)
- Methicillin-susceptible	4	10
- Methicillin-resistant	7	6
*Enterococcus* spp.	6	4
*Streptococcus* spp.	8	10
Coagulase-negative *Staphylococcus* spp.	4	7
Other gram-positives	4	0
Gram-negatives	34 (50.7%)	20 (35.1%)
*Escherichia coli*	8 (11.9%)	4 (7.0%)
*Pseudomonas aeruginosa*	8 (11.9%)	6 (10.5%)
*Proteus* spp.	5	1
*Enterobacter* spp.	5	1
*Klebsiella* spp.	2	5
*Acinetobacter* spp.	1	2
*Serratia* spp.	5	1

## Data Availability

The data presented in this study are available upon request from the corresponding author.
